# Supplementary material for: Examining the predictors of use of sanitary napkins among adolescent girls: A multi-level approach
Source: PLoS One. 2021 Apr 30;16(4):e0250788. doi: 10.1371/journal.pone.0250788 (PMC8087036; doi:10.1371/journal.pone.0250788)
Supplement: Attachment — Submitted filename: PONE-D-20-32727_Manuscript.docx [file pone.0250788.s001.docx]

**Examining the predictors of use of sanitary napkins among adolescent girls: A multi-level approach**

Shekhar Chauhan^1^, Pradeep Kumar^2^, Strong Pillar Marbaniang^2^, Shobhit Srivastava^2^, Ratna Patel^3*^

**^1^** Ph.D. Research Scholar, Department of Population Policies and Programmes, International Institute for Population Sciences, Mumbai, India

^2^ Ph.D. Research Scholar, Department of Mathematical Demography & Statistics, International Institute for Population Sciences, Mumbai, India

^3^ Ph.D. Research Scholar, Department of Public Health and Mortality Studies, International Institute for Population Sciences, Mumbai, India

*** corresponding author:** ratnapatelbhu@gmail.com

Shekhar Chauhan: shekhariips2486@gmail.com

Pradeep Kumar: pradeepmpsiips@gmail.com

Strong Pillar Marbaniang: marbaniangstrong@gmail.com

Shobhit Srivastava: shobhitsrivastava889@gmail.com

Ratna Patel: [ratnapatelbhu@gmail.com](mailto:ratnapatelbhu@gmail.com)

**Examining the predictors of use of sanitary napkins among adolescent girls: A multi-level approach**

**Abstract**

**Background:** This paper aimed to explore the various factors associated with the use of sanitary napkins among adolescents girl in Uttar Pradesh and Bihar.

**Methods:** The study uses information from Understanding the Lives of Adolescents and Young Adults (UDAYA) project survey conducted in Uttar Pradesh and Bihar in 2016. The study sample consisted of 14,625 adolescent girls aged 10-19 years. The study sample was selectedusing a multi-stage systematic sampling design. Multilevel logistic regression (MLR) was used to identify the individual and community level factors associated with the use of sanitary napkins.

**Results:** The results revealed a wide variation in the use of sanitary napkins across the socio-economic and demographic factors. Use of sanitary napkins was significantly higher among adolescents with 8-9 (51.8%) and 10 and more (75.2 %) years of schooling compared to adolescents who had no formal education (26%). The use of sanitary napkins was higher among non-working adolescent girls (52%) than working adolescent girls (39.9%). Estimates from the MLR showed that adolescent girls reporting frequent exposure to mass media (OR=2.15), belonging to the richest wealth quintile (OR=3.65), and whose mothers had 10 or more years of education (OR=2.15) used sanitary napkins more than their counterparts.

**Conclusion:** Given that education, exposure to mass-media and economic status are significantly associated with the use of sanitary napkins, public health policy should focus on mother awareness about menstrual hygiene, and provide sanitary napkin at an affordable price on a large scale, specifically in rural areas. Involve different stakeholder to develop information, education, and communication for promoting the use of sanitary napkins.

**Keywords:** Sanitary napkins, Adolescents girls, Menstrual hygiene, Uttar Pradesh, Bihar.

**Examining the predictors of use of sanitary napkins among adolescent girls: A multi-level approach**

**Introduction**

Menstruation is a normal cycle and a healthy part of girls’ and women’s life [1] that also has social and religious significance attached to it [2]. Hygienic menstrual management consists of using a clean menstrual management material to absorb or collect blood that can be changed in privacy as frequently as necessary for the duration of the menstruation period, use of soap and water to wash the body as required and availability of facilities to dispose of used menstrual management materials [3]. According to Sarma (2018), the benefits of maintaining good hygiene during periods helps to reduce the risk of urinary tract infections, reduce the incidence of rashes in genitals, ensure good reproductive health, and minimize the risk of cervical cancer [4]. Inadequate menstrual hygiene management among adolescent girls (15-19 years ) is a public health concern particularly in low and middle-income countries [5]. India has 355 million menstruating girls and women. Millions of these women and girlsface uncomfortable and dignified experience with menstrual hygiene management [6].

To promote menstrual hygiene practices among rural adolescent girls, the Indian government in 2011 implemented an initiative to increase awareness on menstrual hygiene, access to and use of high-quality sanitary napkins, and safe disposal of sanitary napkins in an environmentally friendly manner [7]. However, reports showed that 90% of the women in India used an unhygienic cloth to manage menstrual period, whereas only 11.2% used hygienic sanitary pads and 3.9% used locally prepared napkins [8]. A qualitative study in rural Haryana, revealed that only 30% of adolescent girls used sanitary napkins, even though 80% of them were aware about them and 79% were motivated to use sanitary napkins [9].

Poor menstrual hygiene practices due to limited accessibility to water and sanitation, lack of privacy, and unaffordability of sanitary pads can result into adverse health outcomes like reproductive tract infections (RTIs) [2,10] and increased absenteeism among adolescent school girls [11]. Untreated RTIs contribute to 10-15% of fetal wastage and 30-50% of prenatal infection. Reproductive tract infections are also associated with the incidence of cervical cancer, HIV/AIDS, infertility, ectopic, pregnancy and a myriad of other symptoms [2]. Qualitative studies report that the fear and humiliation from leakage of blood and body odour result in menstruating girls being absent from school [12].

Gopalan (2019) points out three barriers to adopting of menstrual hygiene practices in India: the lack of awareness about menstruation, a lack of acceptance about the menstrual period and lack of access to quality hygiene products [13]. Reports from a meta-analysis of 138 studies, showed that about half of adolescent girls in India were not aware of the causes of menarche when it started, with only a quarter understanding the source of bleeding [12]. A large study in India revealed that 70% of women cited cost as the main barrier for not using sanitary pads [11]. Other reasons for not using sanitary napkins were problems with disposal of used sanitary napkins [9]. Apart from individual level factors, menstrual hygiene practices are also influenced by some community characteristics such as cultural taboos, their level of education and awareness [14].

This paper aimed to explore the various factors which determine the use of sanitary napkins among adolescent girls in Uttar Pradesh and Bihar. Existing studies on menstrual hygiene practices among adolescent girls in India are based on micro-level data from different pockets of the country and failed to consider the impact of community characteristics on the use of sanitary napkins [9,11]. Community-level characteristics do not merely indicate the distribution of resources and opportunities in a population, they themselves drive interesting and important social dynamics, which cannot be captured using individual characteristics alone [14]. Hence, the pioneering contribution of this study is to establish a relationship between the individual and community level characteristics and the use of sanitary napkins among adolescent girls.

**Methods**

***Data***

The study utilized data from Understanding the Lives of Adolescents and Young Adults (UDAYA) project survey, which was conducted in two Indian states Uttar Pradesh and Bihar in 2016 by Population Council under the guidance of Ministry of Health and Family Welfare, Government of India. The survey collected detailed information on family, media, community environment, assets acquired in adolescence, and quality of transitions to young adulthood indicators. The sample size for Uttar Pradesh and Bihar was 10,350 and 10,350 adolescents aged 10-19 years, respectively. The required sample for each sub-group of adolescents was determined at 920 younger boys, 2,350 older boys, 630 younger girls, 3,750 older girls, and 2,700 married girls in both states. The effective sample size for this study was 14,625 adolescents girls aged 10-19 years. The UDAYA adopted multi-stage systematic sampling design to provide the estimates for states as a whole as well as urban and rural areas of the states.

***Variable description***

***Outcome variable***

The outcome variable was binary in nature defining the use of sanitary napkins coded as 1 “yes” if the girl was using sanitary napkins or 0 “no”. The variable was generated using the question “Girls use different things during menstrual period to prevent bloodstains from becoming evident. What do you use for this?”. The responses were a. locally prepared napkins b. use sanitary napkins c. use cloth d. used nothing e. use other (specify). Responses were recoded as 1 (locally prepared napkins/sanitary napkins) and 0 (use cloth/used nothing/use other (specify)). Additionally in some cases girls were using both napkins and cloths those cases were included as use of sanitary napkins only (less than 1% cases).

***Explanatory variables***

1. Age was coded as 10-12, 13-14,15-17, 18-19 years.
2. Education was coded as “no education”, “1-7 years”, “8-9 years” and “10 or more years”.
3. Working status was coded as “no” and “yes”.
4. Media exposure was coded as “no”, “rare” and “frequent”.
5. Mother’s education was coded as “no education”, “1-7 years”, “8-9 years” and “10 or more years”.
6. Wealth index was coded as “poorest” “poorer” middle” richer” and richest”.
7. Caste was coded as “Scheduled caste/Scheduled tribe (SC/ST)” and “non SC/ST”.
8. Religion was coded as “Hindu” and “non Hindu”.
9. Residence was available in the data as “urban” and “rural”.
10. Survey was conducted in two states “Uttar Pradesh” and “Bihar”.

Community-level variables were constructed by aggregating individual/household-level characteristics of the respondents at the primary sampling unit (PSU) level. The UDAYA data provided a household wealth index (WI) based on information collected on household amenities and assets. The community economic index was divided into two categories, low and high, with low being for PSUs whose average household WI was less than the national average of WI and high being that for the remaining PSUs. Similarly, the individual’s educational index was created based on the average years of schooling of women at the PSU level. A similar index for mother’s education was also created. A community media exposure index was also created based on average media exposure at the PSU level and then dividing it into low and high as per average media exposure.

***Statistical analysis***

We used bivariate analysis (chi-square tests) to examine the association between the outcome variable and other socio-demographic predictors. We employed multilevel logistic regression to assess the effect of the individual-, household (family)-, and community-level variables on the use of sanitary napkin among adolescent girls. The random effects of household and community were estimated by using *melogit* command in STATA (version 15).

The application of the multilevel modeling is justified by the hierarchal structure of the survey, where adolescents were nested within households, and the households were nested within PSUs. We first ran a null model, that is, without keeping any predictors. The null model represented the total variance in the use of sanitary napkin at household and community levels. We then fitted three models, in the first model, we included individual-level predictors. The second model included individual and household level variables. In the final model (Model 3), we added community-level variables in addition to individual and household level predictors. For all the estimated models, the significance of random effects was evaluated by using p-values at a 95% confidence interval.

The mathematical description of the final model (three levels) is given below:

$$logit\left( \pi_{ijk} \right)=log\left( \frac{\pi_{ijk}}{1-\pi_{ijk}} \right)=\beta_{0jk}+\beta_{1}x_{1ijk}+\beta_{2}x_{2ijk}+\beta_{3}x_{3ijk}+\ldots+\beta_{n}x_{nijk}$$

Here, $\pi_{ijk}=p(y_{ijk}=1)$ is the probability that adolescents (i) in the household j, from the PSU k, use a sanitary napkin. Where $y_{ijk}$ is equal to “1” if an adolescent girl uses a sanitary napkin and “0” if she did not. The study defined this probability as a function of an intercept and the exploratory variables as follows:$\beta_{0jk}=\beta_{0}+\mu_{0jk}$.

In this equation, β_0jk_ indicates that the paper modeled the intercept in this relationship as random at $j^{th}$ (household) and $k^{th}$ (PSU) levels. The variables $x_{1ijk}to x_{nijk}$ were the exploratory variables, and their coefficients were fixed effects. The technical advantage of this methodology relies on the error term structure. Linear or logistic regression models exhibit one error term for the whole equation, whereas multilevel analysis generates one error term for each level, isolating the individual-level and group-level residual variance. The split error term in the multilevel analysis allows assessing unobserved effects at every level [16].

**Results**

The socio-demographic profile of adolescent girls was presented in *Table 1*. The majority of teenage girls were aged 15-19 years (88.7%), about one-third of adolescents had 10 or more years of schooling, and only 16.7 per cent were working. Around half of the adolescents reported frequent exposure to mass media, and three-fourths of girls’ mothers had no schooling. One-fourth of adolescent girls belonged to scheduled caste/scheduled tribe group, 78.5 per cent were Hindu, and about 84 per cent lived in rural areas.

| **Table-1** **Socio-demographic profile of adolescent girls** | | |
| --- | --- | --- |
| **Variables** | **Sample** | **Percentage** |
| **Age (years)** |  |  |
| Early adolescents (10-14) | 1,653 | 11.3 |
| Late adolescents (15-19) | 12,972 | 88.7 |
| **Education (years)** |  |  |
| No education | 1,890 | 12.9 |
| 1-7 | 3,939 | 26.9 |
| 8-9 | 4,093 | 28.0 |
| 10 or more | 4,703 | 32.2 |
| **Working status** |  |  |
| No | 12,179 | 83.3 |
| Yes | 2,446 | 16.7 |
| **Media exposure** |  |  |
| No exposure | 2,703 | 18.5 |
| Rarely | 4,212 | 28.8 |
| Frequently | 7,710 | 52.7 |
| **Mother's education (years)** |  |  |
| No education | 10,946 | 74.9 |
| 1-7 | 1,476 | 10.1 |
| 8-9 | 986 | 6.7 |
| 10 or more | 1,216 | 8.3 |
| **Wealth index** |  |  |
| Poorest | 1,971 | 13.5 |
| Poorer | 2,735 | 18.7 |
| Middle | 3,188 | 21.8 |
| Richer | 3,577 | 24.5 |
| Richest | 3,154 | 21.6 |
| **Caste** |  |  |
| SC/ST | 3,784 | 25.9 |
| Non-SC/ST | 10,841 | 74.1 |
| **Religion** |  |  |
| Hindu | 11,479 | 78.5 |
| Non-Hindu | 3,146 | 21.5 |
| **Residence** |  |  |
| Urban | 2,356 | 16.1 |
| Rural | 12,269 | 83.9 |
| **State** |  |  |
| Uttar Pradesh | 9,855 | 67.4 |
| Bihar | 4,770 | 32.6 |
| **Total** | 14,625 | 100.0 |
| *SC/ST: Scheduled caste/Scheduled tribe* | | |

Use of sanitary napkins among adolescent girls by background characteristics are presented in Table 2. The use of sanitary napkin was significantly higher among late adolescents (54.1 per cent), and it was low among early adolescent girls (17.3 per cent). A higher proportion of adolescents with 8-9 (51.8 per cent) and 10 or more (75.2 per cent) years of schooling than those who were uneducated (26 per cent) used sanitary napkins. The use of sanitary napkin was higher among adolescents who were not working (52 per cent) than those who were working (39.9 per cent). Moreover, the use of sanitary napkin was more prevalent among adolescents who reported frequent exposure to mass media (62.8 per cent), and it was lowest among those who had no exposure to media (24.6 per cent). Mother’s education and wealth index were significantly positively associated with the use of sanitary napkins. A higher proportion of girls whose mothers had 10 or more years of education (81.2 percent) used sanitary napkins than those whose mothers had no education (43.9 per cent). The proportion of girls using sanitary napkins was higher among girls from the richest households than those from poorer households. Moreover, the use of sanitary napkins was lower among SC/ST (43.6 per cent) and non-Hindu (46.6 per cent) adolescents compared to non-SC/ST (52.2 per cent) and Hindu adolescents (50.9 per cent). The use of sanitary napkins was significantly higher among urban adolescents than rural ones.

| **Table-2 Percentage distribution of adolescent girls who use sanitary napkins by background characteristics** | | |
| --- | --- | --- |
| **Variables** | **Percentage** | **P<0.05** |
| **Age (years)** |  | * |
| Early adolescents (10-14) | 17.3 |  |
| Late adolescents (15-19) | 54.1 |  |
| **Education (years)** |  | * |
| No education | 26.0 |  |
| 1-7 | 29.4 |  |
| 8-9 | 51.8 |  |
| 10 and above | 75.2 |  |
| **Working status** |  | * |
| No | 52.0 |  |
| Yes | 39.9 |  |
| **Media exposure** |  | * |
| No exposure | 24.6 |  |
| Rarely | 42.8 |  |
| Frequently | 62.8 |  |
| **Mother's education (years)** |  | * |
| No education | 43.9 |  |
| 1-7 | 57.7 |  |
| 8-9 | 66.8 |  |
| 10 and above | 81.2 |  |
| **Wealth index** |  | * |
| Poorest | 27.5 |  |
| Poorer | 32.8 |  |
| Middle | 45.1 |  |
| Richer | 56.1 |  |
| Richest | 76.8 |  |
| **Caste** |  | * |
| SC/ST | 43.6 |  |
| Non-SC/ST | 52.2 |  |
| **Religion** |  |  |
| Hindu | 50.9 |  |
| Non-Hindu | 46.6 |  |
| **Residence** |  | * |
| Urban | 68.3 |  |
| Rural | 46.4 |  |
| **State** |  |  |
| Uttar Pradesh | 51.6 |  |
| Bihar | 46.7 |  |
| **Total** | 50.0 |  |
| *SC/ST: Scheduled caste/Scheduled tribe; *if p<0.05* | | |

The estimates from multilevel logistic regression analysis, showing the odds ratios with 95% confidence interval of factors associated with the use of the sanitary napkin, are presented in ***Table 3***. Model 1 included individual-level predictor variables. Model 2 included household-level variables in addition to the explanatory variables used in Model 1 and Model 3 added community-level variables. In Model 1, age, adolescents’ educational level, working status, mass media exposure, and mother’s education status were significantly associated with the use of sanitary napkins. Model 3 revealed that the use of sanitary napkin was 6.82 times significantly higher among late adolescents (OR: 6.82; CI: 5.36-8.67) than early adolescents. The likelihood of sanitary napkin use was 1.41, 3.10, and 6.51 times higher among adolescents with 1-7 (OR: 1.41; CI: 1.20-1.67), 8-9 (OR: 3.10; CI: 2.57-3.74), and 10 or more (OR: 6.51; CI: 5.15-8.23) years of schooling compared to adolescents with no education. The odds of sanitary napkin use were 20 percent lower among working adolescents (OR: 0.80; CI: 0.70-0.92) than those who were not working. Moreover, adolescents who reported rare (OR: 1.43; CI: 1.22-1.67) or frequent (OR: 2.15; CI: 1.81-2.54) exposure to mass media were significantly more likely to use sanitary napkins compared to those who had no exposure to mass media. Mother’s education has a significant effect on the use of sanitary napkin among adolescent girls.

The likelihood of sanitary napkin use was significantly higher among girls from middle (OR: 1.51; CI: 1.26-1.81), richer (OR: 1.79; CI: 1.48-2.17), and richest (OR: 3.65; CI: 2.87-4.64) households compared to poorest ones. In comparison to SC/ST adolescents, non-SC/ST adolescents had higher odds of sanitary napkin use (OR: 1.22; CI: 1.08-1.39).

Adolescents from communities with a higher education index (OR: 1.09; CI: 0.87-1.28) had higher odds of sanitary napkin use than adolescents from communities with low levels of education, though the result was not significant. Surprisingly, adolescents from communities with high media exposure (OR: 0.73; CI: 0.57-0.93) were 27 per cent less likely to use sanitary napkins than those from communities with low media exposure. Moreover, adolescents from rural areas were 38 per cent less likely (OR: 0.62; CI: 0.49-0.78) to use sanitary napkins than those from urban areas.

| **Table-3** **Multilevel logistic regression analysis assessing the effect of background characteristics on the likelihood of use of sanitary napkins among adolescents** | | | |
| --- | --- | --- | --- |
| **Variables** | **Model-1** | **Model-2** | **Model-3** |
| **Age (years)** | **OR (95%CI)** | **OR (95%CI)** | **OR (95%CI)** |
| Early adolescents (10-14) | Ref. | Ref. | Ref. |
| Late adolescents (15-19) | 6.32*(5,7.98) | 6.87*(5.4,8.75) | 6.82*(5.36,8.67) |
| **Education (years)** |  |  |  |
| No education | Ref. | Ref. | Ref. |
| 1-7 | 1.44*(1.22,1.70) | 1.39*(1.18,1.64) | 1.41*(1.20,1.67) |
| 8-9 | 3.37*(2.79,4.07) | 3.02*(2.51,3.64) | 3.10*(2.57,3.74) |
| 10 and above | 7.90*(6.20,10.05) | 6.30*(4.99,7.94) | 6.51*(5.15,8.23) |
| **Working status** |  |  |  |
| No | Ref. | Ref. | Ref. |
| Yes | 0.69*(0.60,0.79) | 0.79*(0.68,0.91) | 0.80*(0.70,0.92) |
| **Media exposure** |  |  |  |
| No exposure | Ref. | Ref. | Ref. |
| Rarely | 1.53*(1.31,1.79) | 1.46*(1.25,1.71) | 1.43*(1.22,1.67) |
| Frequently | 3.14*(2.62,3.76) | 2.35*(1.98,2.79) | 2.15*(1.81,2.54) |
| **Mother's education (years)** |  |  |  |
| No education | Ref. | Ref. | Ref. |
| 1-7 | 1.54*(1.3,1.82) | 1.37*(1.16,1.62) | 1.34*(1.14,1.59) |
| 8-9 | 1.99*(1.62,2.45) | 1.68*(1.37,2.07) | 1.64*(1.33,2.02) |
| 10 and above | 3.11*(2.5,3.86) | 2.26*(1.83,2.8) | 2.15*(1.74,2.65) |
| **Wealth index** |  |  |  |
| Poorest |  | Ref. | Ref. |
| Poorer |  | 1.08(0.9,1.3) | 1.07(0.9,1.29) |
| Middle |  | 1.58*(1.32,1.9) | 1.51*(1.26,1.81) |
| Richer |  | 1.95*(1.6,2.37) | 1.79*(1.48,2.17) |
| Richest |  | 4.08*(3.19,5.21) | 3.65*(2.87,4.64) |
| **Caste** |  |  |  |
| SC/ST |  | Ref. | Ref. |
| Non-SC/ST |  | 1.24*(1.09,1.41) | 1.22*(1.08,1.39) |
| **Religion** |  |  |  |
| Hindu |  | Ref. | Ref. |
| Non-Hindu |  | 0.94(0.81,1.09) | 0.92(0.79,1.07) |
| **Community wealth index** |  |  |  |
| Low |  |  | Ref. |
| High |  |  | 0.89(0.70,1.14) |
| **Community education index** |  |  |  |
| Low |  |  | Ref. |
| High |  |  | 1.09(0.87,1.28) |
| **Community media index** |  |  |  |
| Low |  |  | Ref. |
| High |  |  | 0.73*(0.57,0.93) |
| **Community education (mother)** |  |  |  |
| Low |  |  | Ref. |
| High |  |  | 0.81(0.67,1.03) |
| **Residence** |  |  |  |
| Urban |  |  | Ref. |
| Rural |  |  | 0.62*(0.49,0.78) |
| **State** |  |  |  |
| Uttar Pradesh |  |  | Ref. |
| Bihar |  |  | 1.40*(1.17,1.67) |
| *SC/ST: Scheduled caste/Scheduled tribe; *if p<0.05; OR: Odds Ratio; CI: Confidence Interval* | | | |

A null model (without covariates) for the use of sanitary napkins among adolescent girls (***Table 4***) revealed a significant amount of variation across households and communities. Based on intra-class correlation coefficient (ICC) values, 38 per cent and 20 per cent of the total variance in the use of sanitary napkins among adolescent girls were attributable to differences across families and communities, respectively. After including individual- (Model 1), household- (Model 2), and community-level variables (Model 3) in the null model, the ICC values decreased to 7 per cent (community level) and 30 per cent (household level). The results suggest that the likelihood of sanitary napkin use was influenced by a similar decision of another adolescent girl from the same household and/or community.

| **Table 4: Variance estimates across families and communities, and intra-class correlation coefficient for the multilevel models for use of sanitary napkin among adolescent girls** | | | | |
| --- | --- | --- | --- | --- |
| **Random Effect Parameters** | **Null** | **Model 1** | **Model 2** | **Model 3** |
| Community (PSU) random variance (SE) | 1.04 (0.131) | 0.617 (0.088) | 0.497 (0.073) | 0.327 (0.051) |
| Household random variance (SE) | 0.958 (0.269) | 1.08 (0.323) | 1.11 (0.331) | 1.09 (0.329) |
| Community (PSU) ICC (%) | 0.20 | 0.12 | 0.10 | 0.07 |
| Household ICC (%) | 0.38 | 0.34 | 0.33 | 0.30 |
| SE: standard error; PSU: primary sampling unit; ICC: intra-class correlation coefficient | | | | |

**Discussion:**

Even though this study focused on adolescent girls only, the study adds relevant information to available literature. The use of sanitary napkin is essential as studies have noted that poor menstrual hygiene may promote reproductive tract infections among [8]. In this study we examined the predictors of sanitary napkin use among adolescent girls by adopting a multi-level approach. For a comprehensive analysis, we examined four important community-level variables, namely, community wealth index, community education index, community media index, and community education index, along with individual and household level variables. We identified several predictors of the use of sanitary napkins among adolescent girls.

Adolescents aged 15-19 years were more likely to use sanitary napkins than those aged 10-14 years. Older adolescents tend to have high levels of education, which may explain their higher use of sanitary napkins as we found that adolescents with higher education were more likely to use sanitary napkin than those who had no education. Other scholars have acknowledged the importance of education in promoting sanitary use among adolescent girls [17]. Education among adolescents girls promotes their mass-media exposure, which further promotes sanitary use among them [18]. The relationship between sanitary napkin and education is bi-direction as the use of sanitary napkins may also promote girls’ education [19]. Montgomery et al., in their study, noted that the distribution of sanitary napkins improves school attendance among girls [19]. The results from multi-level analysis further noted that a high level of community education is associated with higher use of sanitary napkins among adolescent girls. However, the finding was not significant. A study has noted the importance of community health education in bringing improvements in the utilization of hygienic practices during menstruation [2].

Media exposure among adolescent girls is one of the important predictors of sanitary use among them. We found that adolescents who were exposed to media were more likely to use sanitary napkin than adolescents who had no exposure to media. Similar findings have been reported elsewhere [20-22]. Mass-media is a major source of information about menstrual hygiene; therefore, girls with frequent mass-media exposure may tend to have higher sanitary napkin use than their counterparts [23]. However, results related to media exposure from the community-level analysis showed a different picture. Results noted that high community media index was associated with low use of sanitary napkin among adolescent girls.

Higher levels of mother’s education were associated with higher use of sanitary napkin among adolescent girls. This finding is in agreement with previously available literature [21]. However, the association between community education (mother) and the use of sanitary napkin was not significant. Mothers play a key role in educating their daughters about health matters [10,21]. Having an educated mother can therefore play a significant role in maintaining menstrual hygiene [23,24]. Furthermore, the importance of mother’s education was outlined by the fact that girls whose mothers were uneducated were more likely to miss school during their menstruation than girls whose mothers were educated [25].

The wealth index of the household is another predictor of the use of sanitary napkin among adolescents. The study found that a higher wealth index was associated with higher use of sanitary napkins among adolescent girls. A previous study also concluded that the use of sanitary napkins was higher among adolescents in rich households than among adolescents in poor households [26]. Adolescents are dependent on their parents to buy them sanitary napkins;as such, poor parents may find it difficult purchase sanitary napkins for their daughters [27] [28].

We found that the use of sanitary napkin was higher among non-SC/ST adolescents than their counterparts. In the Indian context, SC/ST tend have poorer socio-economic status, which may limit the use of sanitary napkins among them [29]. Furthermore, we found that the use of sanitary napkin was higher among adolescent girls in urban areas than in rural areas. Similar findings have been reported elsewhere [8,30,31]. Higher use of sanitary napkins among urban girls may be attributed to their higher level of education, better school infrastructure, and easy access to sanitary napkins. Narayan et al. suggested that urban girls have a higher level of awareness about hygienic menstrual practices than their rural counterparts [32]. Further, as noted by Kathuria and Raj rural women have limited access to toilets and tend to defecate in the openand do not wear undergarments [31]. As such, they would be unable to use sanitary napkins, which can only be used with undergarments [31]. Moreover, in rural areas, girls may avoid buying sanitary napkins from shops with male shopkeepers due to shame [31]. Furthermore, disposing of sanitary napkins is another issue in the rural area, and therefore rural women find cloth a comfortable medium to use during menstruation [33].

Despite providing in-depth information about the predictors of the use of sanitary napkin among adolescent girls, the study has some limitations. The study was conducted only in two of the Indian states, namely, Uttar Pradesh and Bihar; therefore, the findings are not be generalizeable to the country population.

**Conclusion:**

There is an urgent need to promote the use of sanitary napkin during menstruation; the need is important not only from the public health point of view but also for assisting adolescent girls in realizing their full potential. Although India’s prime minister Shri Narendra Modi in his 74^th^ Independence Day speech on 15^th^ August 2020, stressed the importance of sanitary napkins, current national programs do not lay emphasis on menstrual hygiene. Mothers are an important source of knowledge about menstrual hygiene to their daughters. Therefore, mothers should be counseled to discuss menstruation issues with their daughters before the age of menarche. Mothers should be informed about the ill-effects of clinging to outlandish taboos on menstruation. Furthermore, there is a need to provide sanitary napkins at an affordable price on a large scale, especially in rural areas. Involving different stakeholders to promote the use of sanitary napkins among adolescent girls is important. Moreover, improvements in girl’s education will certainly help in promoting the use of sanitary napkin among them.

**Acknowledgement:** Not applicable

**Funding:** This research did not receive any specific grant from funding agencies in the public, commercial, or not-for-profit sectors.

**Declaration of Interests:** None

**Author’s Contribution:**

**Conceptualization:** Pradeep Kumar, Strong Pillar Marbaniang, Shobhit Srivastava, Shekhar Chauhan, Ratna Patel

**Formal Analysis:** Pradeep Kumar, Shobhit Srivastava

**Investigation:** Pradeep Kumar, Shobhit Srivastava, Shekhar Chauhan, Ratna Patel

**Methodology:** Pradeep Kumar, Shobhit Srivastava

**Supervision:** Pradeep Kumar, Strong Pillar Marbaniang, Shobhit Srivastava, Shekhar Chauhan, Ratna Patel

**Writing Original Draft:** Shekhar Chauhan, Strong Pillar Marbaniang, Ratna Patel

**Revising and Finalizing the draft manuscript:** Pradeep Kumar, Strong Pillar Marbaniang, Shobhit Srivastava, Shekhar Chauhan, Ratna Patel

**References:**

1. Singh AJ. Place of menstruation in the reproductive lives of women of rural North India. Indian Journal of Community Medicine. 2006 Jan 1;31(1):10. <https://doi.org/10.4103/0970-0218.54923>
2. Garg R, Goyal S, Gupta S. India moves towards menstrual hygiene: subsidized sanitary napkins for rural adolescent girls—issues and challenges. Maternal and child health journal. 2012 May 1;16(4):767-74. https://doi.org/10.1007/s10995-011-0798-5
3. Sommer M, Sahin M. Overcoming the taboo: advancing the global agenda for menstrual hygiene management for schoolgirls. American journal of public health. 2013 Sep;103(9):1556-9. https://doi.org/10.2105/AJPH.2013.301374
4. Sarma, B., 2018. Importance of Maintaining Good Hygiene During Periods [WWW Document]. heyday. URL https://www.heydaycare.com/importance-of-maintaining-good-hygiene-during-periods/ (accessed 9.4.20).
5. Sharma S, Mehra D, Brusselaers N, Mehra S. Menstrual Hygiene Preparedness Among Schools in India: A Systematic Review and Meta-Analysis of System-and Policy-Level Actions. International journal of environmental research and public health. 2020 Jan;17(2):647. https://doi.org/10.3390/ijerph17020647
6. Alexandra, G., Iyer, L., Kasen, P., Mazzola, F., Peterson, K., 2016. Menstrual Health in India: Country Landscape Analysis [WWW Document]. URL https://menstrualhygieneday.org/wp-content/uploads/2016/04/FSG-Menstrual-Health-Landscape_India.pdf (accessed 9.5.20).
7. National Health Mission (NHM), 2011. MENSTRUAL HYGIENE SCHEME(MHS) [WWW Document]. URL https://nhm.gov.in/index1.php?lang=1&level=3&sublinkid=1021&lid=391 (accessed 9.9.20).
8. Anand E, Singh J, Unisa S. Menstrual hygiene practices and its association with reproductive tract infections and abnormal vaginal discharge among women in India. Sexual & Reproductive Healthcare. 2015 Dec 1;6(4):249-54. <https://doi.org/10.1016/j.srhc.2015.06.001>
9. Bhattacharya S, Singh A. How effective is the Menstrual Hygiene Scheme? An evaluation study from North India. Int J Community Med Public Health. 2016 Sep;3:2584-6. https://doi.org/10.18203/2394-6040.ijcmph20163077
10. Dasgupta A, Sarkar M. Menstrual hygiene: how hygienic is the adolescent girl?. Indian journal of community medicine: official publication of Indian Association of Preventive & Social Medicine. 2008 Apr;33(2):77. https://doi.org/[10.4103/0970-0218.40872](https://dx.doi.org/10.4103%2F0970-0218.40872)
11. Shah SP, Nair R, Shah PP, Modi DK, Desai SA, Desai L. Improving quality of life with new menstrual hygiene practices among adolescent tribal girls in rural Gujarat, India. Reproductive Health Matters. 2013 Jan 1;21(41):205-13. https://doi.org/10.1016/S0968-8080(13)41691-9
12. Van Eijk AM, Sivakami M, Thakkar MB, Bauman A, Laserson KF, Coates S, Phillips-Howard PA. Menstrual hygiene management among adolescent girls in India: a systematic review and meta-analysis. BMJ open. 2016 Mar 1;6(3). https://doi.org/10.1136/bmjopen-2015-010290
13. Gopalan, M., (2019). There are 3 barriers blocking good menstrual hygiene for all women. Here’s how we overcome them [WWW Document]. World Econ. Forum. URL https://www.weforum.org/agenda/2019/11/menstruation-in-different-cultures-period-taboos/ (accessed 9.5.20).
14. Lahme AM, Stern R, Cooper D. Factors impacting on menstrual hygiene and their implications for health promotion. Global health promotion. 2018 Mar;25(1):54-62. https://doi.org/10.1177/1757975916648301
15. Colleran H, Jasienska G, Nenko I, Galbarczyk A, Mace R. Community-level education accelerates the cultural evolution of fertility decline. Proceedings of the Royal Society B: Biological Sciences. 2014 Mar 22;281(1779):20132732. https://doi.org/10.1098/rspb.2013.2732
16. Kumar P, Dhillon P. Household-and community-level determinants of low-risk Caesarean deliveries among women in India. Journal of Biosocial Science. 2020 Jan 30:1-6. https://doi.org/10.1017/S0021932020000024
17. Nitika PL. Prevalence and determinants of menstrual disorders and napkin usage among women in India using DLHS-4 data. Journal of family medicine and primary care. 2019 Jun;8(6):2106. https://doi.org/[10.4103/jfmpc.jfmpc_262_19](https://dx.doi.org/10.4103%2Fjfmpc.jfmpc_262_19)
18. Ramaiya A, Malhotra A, Cronin C, Stevens S, Kostizak K, Sharma A, Nagar S, Sood S. How does a social and behavioral change communication intervention predict menstrual health and hygiene management: a cross-sectional study. BMC public health. 2019 Dec 1;19(1):1039. [https://doi.org/10.1186/s12889-019-7359-z](about:blank)
19. Montgomery P, Ryus CR, Dolan CS, Dopson S, Scott LM. Sanitary pad interventions for girls' education in Ghana: a pilot study. PloS one. 2012 Oct 31;7(10):e48274. <https://doi.org/10.1371/journal.pone.0048274>
20. Khanna A, Goyal RS, Bhawsar R. Menstrual practices and reproductive problems: a study of adolescent girls in Rajasthan. Journal of health management. 2005 Apr;7(1):91-107. [https://doi.org/10.1177/097206340400700103](https://doi.org/10.1177%2F097206340400700103)
21. Sudeshna R, Aparajita D. Determinants of menstrual hygiene among adolescent girls: a multivariate analysis. Natl J Community Med. 2012 Apr;3(2):294-301. <http://njcmindia.org/uploads/3-2_294-301.pdf>
22. Goli S, Sharif N, Paul S, Salve PS. Geographical disparity and socio-demographic correlates of menstrual absorbent use in India: A cross-sectional study of girls aged 15–24 years. Children and Youth Services Review. 2020 Oct 1;117:105283. <https://doi.org/10.1016/j.childyouth.2020.105283>
23. El-Gilany AH, Badawi K, El-Fedawy S. Menstrual hygiene among adolescent schoolgirls in Mansoura, Egypt. Reproductive health matters. 2005 Jan 1;13(26):147-52. <https://doi.org/10.1016/S0968-8080(05)26191-8>
24. Deo DS, Ghattargi CH. Perceptions and practices regarding menstruation: a comparative study in urban and rural adolescent girls. Indian J Community Med. 2005 Jan;30(1):33.
25. Vashisht A, Pathak R, Agarwalla R, Patavegar BN, Panda M. School absenteeism during menstruation amongst adolescent girls in Delhi, India. Journal of family & community medicine. 2018 Sep;25(3):163. https://doi.org/[10.4103/jfcm.JFCM_161_17](https://dx.doi.org/10.4103%2Fjfcm.JFCM_161_17)
26. Sharma S, Mehra D, Kohli C, Singh MM. Menstrual hygiene practices among adolescent girls in a resettlement colony of Delhi: a cross-sectional study. Int J Reprod Contracept Obstet Gynecol. 2017;6(5):1945-51. http://dx.doi.org/10.18203/2320-1770.ijrcog20171954
27. Anchebi H, Shiferaw B, Fite R, Abeya S. Practice of menstrual hygiene and associated factors among female high school students in adama town. J Women's Health Care. 2017;6(370):2167-0420. https://doi.org/10.4172/2167-0420.1000370
28. Kaur R, Kaur K, Kaur R. Menstrual hygiene, management, and waste disposal: Practices and challenges faced by girls/women of developing countries. Journal of environmental and public health. 2018 Feb 20;2018.
29. Rani P. Knowledge and practices of menstrual hygiene among married adolescents and young women in chittoor district of Andra Pradesh: India. J Nurs Health Sci. 2014;3(2):06-15.
30. Paria B, Bhattacharyya A, Das S. A comparative study on menstrual hygiene among urban and rural adolescent girls of West Bengal. Journal of family medicine and primary care. 2014 Oct;3(4):413. https://doi.org/[10.4103/2249-4863.148131](https://dx.doi.org/10.4103%2F2249-4863.148131)
31. Kathuria B, TP SR. Effects of Socio-Economic Conditions on Usage of Hygienic Method of Menstrual Protection among Young Women in EAG States of India. 2018. <https://amity.edu/UserFiles/admaa/0d862Paper%204.pdf>
32. Narayan K, Srinivasa DK, Pelto PJ, Veerammal S. Puberty rituals, reproductive knowledge and health of adolescent schoolgirls in South India. Asia-Pacific Population Journal. 2001 Mar 31;16(2):225-38. <https://doi.org/10.18356/2702b8d0-en>
33. Chakravarty D. Fighting the Menstrual Hygiene battle in rural India: A development communication perspective of the menstrual practices of rural India. Journal of Content, Community & Communication. 2016;4(2):26-32.
