# Supplementary material for: Examining the predictors of use of sanitary napkins among adolescent girls: A multi-level approach
Source: PLoS One. 2021 Apr 30;16(4):e0250788. doi: 10.1371/journal.pone.0250788 (PMC8087036; doi:10.1371/journal.pone.0250788)
Supplement: Attachment — Submitted filename: Review comments PONE-D-20-32727.docx [file pone.0250788.s002.docx]

**Title: Examining the predictors of use of sanitary napkins among adolescent girls: A multilevel approach**

**Review comments to the author**

**Summary of the research**

Authors have tried to establish linkages between individual, household and community level factors on the use of sanitary napkins by young girls. The research topic is interesting and of high issue for girls of lower- and middle-income countries. Overall, the study is well designed and written in lucid and clear language, however writing quality could have improved to some extent to bring more clarity. I observed that somewhere the authors have used tenses casually or given incomplete sentences/recommendation making the text unclear to readers.

**Abstract:**

1. The authors state that ‘Involve different stakeholder…….sanitary napkins’. The statement doesn’t clarify, who should involve stakeholders – program implementing partner or government or NGOs?? It should be stated clearly for whom the recommendations are made for.

**Introduction:**

1. The first paragraph of the introduction section mentioned that ‘According to Sarma, (2018), ….. unitary tract infections,….. cervical cancer. Authors should write carefully about the infections quoted from other studies. Here, ‘…unitary tract infection…’ has no meaning and diverting the relevance of the topic.
2. Authors should introduce abbreviations before using it repeatedly. For eg. In the second paragraph of introduction, RTI is used several time without being mention what does it stands for.
3. In the sentence ‘Increasingly RTIs….ectopic, pregnancy …..symptoms.’; ectopic pregnancy are used together without the use of comma ( , )

**Figures and tables:**

1. Authors have used different styles for writing Table titles: for eg. Table-1, Table-2, Table-3, Table 4: , somewhere title indent is in middle, somewhere it is in left. One should use uniform style as preferred by the journal.
2. Figure in tables should use uniform number of decimals, some places figure in tables/ confidence intervals used whole number, somewhere it is in 2 decimals. Please make them uniform

**Methods:**

1. In the data section, the author has written ‘The required sample….in both states.’ The author however has not explained the age group to differentiate the younger boys/girls from older boys/girls. Also, the sample of 2700 married girls are not clear if these married girls’ sample are included in younger/older age group or excluded and made separate category irrespective of the younger/older category.
2. Authors can add a section on limitations of the research, if any.

**Results:**

1. Author has casually used the tenses in result section. For eg: while describing Table 3 author has written ‘Model 1 includes individual-level…..’ and further Model 2 included household-level…’ ; ‘Mother’s education has a significant……’. Use of tenses should be used appropriately.

**Discussions:**

1. The sentence is not clear ‘The use of sanitary napkin …….. reproductive tract infections among’. This sentence is incomplete as a reader cannot understand who the audience are? Among whom?
2. Author has casually written community education index twice in the sentence ‘For a comprehensive analysis….household level variables’.

**Conclusion:**

1. Author has written ‘Furthermore, there is a need to provide sanitary napkin at an affordable price on a large scale, specifically in rural areas’. Authors should be careful while writing such incomplete recommendation. This statement is not clear to me, who should provide sanitary napkins? What is the rationale behind who should provide sanitary napkins?

.
