# Supplementary material for: Examining the predictors of use of sanitary napkins among adolescent girls: A multi-level approach
Source: PLoS One. 2021 Apr 30;16(4):e0250788. doi: 10.1371/journal.pone.0250788 (PMC8087036; doi:10.1371/journal.pone.0250788)
Supplement: Attachment — Submitted filename: Response to reviewers.docx [file pone.0250788.s004.docx]

**Editor’s comments:**

Please review your reference list to ensure that it is complete and correct. If you have cited papers that have been retracted, please include the rationale for doing so in the manuscript text, or remove these references and replace them with relevant current references. Any changes to the reference list should be mentioned in the rebuttal letter that accompanies your revised manuscript. If you need to cite a retracted article, indicate the article’s retracted status in the References list and also include a citation and full reference for the retraction notice.

**Response:** The authors have reviewed the reference list. It is correct. There are 40 references in manuscript text as well as in the reference section. Also, authors have checked for the authenticity of the references. None of the papers cited in the manuscript have been retracted.

----------------------------------------------------------------------------------------------------------------

Reviewer #2: The authors have done a good job addressing the reviewers’ comments. Below are some outstanding issues that need to be addressed.

**Response:** The authors are thankful to the reviewer for revising the manuscript critically and for providing the comments.

**Abstract**
The word “examining” in the first sentence should be replaced with “associated with”
Add a space between “(UDAYA)” and “project” The abstract conclusion is unclear and needs to be revised. Essentially, the authors found that education, exposure to mass media and economic status were significantly associated with the use of sanitary towels and propose that “it is important to bring improvements” in these indicators. However, more reflection on these findings and their implications is needed. For example, the association between economic status and sanitary towel use, suggests that the cost of sanitary towels might be prohibitive for some girls and that policies and programs that make sanitary products more financially accessible may be warranted. In the same vein, it is unclear what the authors mean by “improve mass media exposure among girls.”

**Response:** The abstract has been amended as suggested. The conclusion section has been updated on the suggested lines.

**Introduction**
Paragraph 1: Revise the following sentence…” The benefits of maintaining good hygiene during periods help to reduce the risk of urinary tract infections, reduce the incidence of rashes in genitals, ensure good reproductive health, and minimize the risk of cervical cancer [3,4,5].” To “The benefits of maintaining good hygiene during periods include a reduced risk of urinary tract infections, genital rashes, and cervical cancer [3,4,5].”
Paragraph 3: There is no need to capitalize ‘Reproductive Tract Infections’
Last paragraph: Revise first sentence – “This paper aimed to explore the factors associated with the use of sanitary napkins…” The framing is important – in a cross-sectional study you cannot assess the factors that DETERMINE sanitary napkin use, you can only speak of associations.

**Response:** The introduction part has been modified as per suggestions provided by the reviewer.

**Methods (data)**

Paragraph 1: add a comma before “Uttar Pradesh and Bihar” in the first sentence

**Response:** The correction has been made as suggested.

Paragraph 1: revise second to last sentence to remove the word “in” before “ages”. The word appears several times

**Response:** The correction has been made as suggested.

Paragraph 2: the acronym PSU is not defined Last paragraph: Correct “About 609 girls (4.2%) were excluded from the sample because they had not started menstruating.” Last paragraph: delete the last sentence, which is repetitive given the next text additions

**Response:** The correction has been made as suggested.

Methods (explanatory variables) Revise the description of media exposure ‘Media exposure assessed the extent to which the respondent was exposed to television, radio, or newspapers. The media exposure variable was coded as “no,” “rare,” and “frequent.”’

**Response:** The correction has been made as suggested.

It may be better to have a separate paragraph to explain the wealth index and to clarify that the scores were then summed up and divided into five quintiles.
Remove comma after “untouchables” and correct to “…that is socially and financially segregated by their low status…”

**Response:** The correction has been made as suggested.

**Results**
Paragraph 1: Correct “…adolescent girls IS presented…”

**Response:** The correction has been made as suggested.

Table 1, seems that the font of the sample and percentage columns is different. Similar use of different font types noted in Table 2, Table 3, and Table 4.

**Response:** The font has been corrected as suggested. Now, the same font has been used in all the tables.

Paragraph 2: insert space between “higher” and “among”

**Response:** The correction has been made as suggested.

Paragraph 3: insert space between “were” and “22”

**Response:** The correction has been made as suggested.

**Discussion**
Paragraph 3: insert space between “elsewhere” and the cited references

**Response:** The correction has been made as suggested.

Paragraph 6: Merge and correct sentence “In India, sanitary pads are sold in chemists and departmental stores, which are more common in urban areas [38].” Delete the next sentence

**Response:** The correction has been made as suggested.

**Limitations**
Correct “Further, the data ARE cross-sectional and causal inferences cannot be made.”

**Response:** The correction has been made as suggested.

**Conclusion**
The conclusion should be revised. Sanitary napkins are just one product – there are tampons, menstrual cups etc., which are considered hygienic products. I would suggest that the authors reframe this as “Ensuring that adolescent girls have access to hygienic means to manage their menses is critical from a public health perspective and in enabling them to realize their full potential. Although……do not emphasize menstrual hygiene. Programs to enhance menstrual hygiene are warranted. These program should involve mothers, who are an important source of knowledge about menstrual hygiene. Facilitating girls’ access to education may also produce tangible menstrual hygiene benefits.” The same conclusion could also be adapted for the abstract

**Response:** The authors are thankful to the reviewer for providing the valuable suggestions related to conclusion. Authors have accordingly updated the conclusion as suggested by the reviewer.

----------------------------------------------------------------------------------------------------------------

Reviewer #3: The basis of sample size calculation has not been explained, what was the reference and formula to calculate the sample size, explain.

**Response:** This study is based on secondary data conducted by Population Council and team and authors did not calculated sample size. Therefore sample size calculation and formula has not given in the manuscript. More details about sampling design and survey methodology are published elsewhere (Santhya et al., 2017) and cited in the manuscript.

1. Santhya KG, Acharya R, Pandey N, Gupta A, Rampal S, Singh S, Zavier AF. Understanding the lives of adolescents and young adults (UDAYA) in Uttar Pradesh, India. New Delhi. 2017. [https://www.popcouncil.org/uploads/pdfs/2017PGY_UDAYA-UPreport.pdf](about:blank)
